# Mechanical behavior and reloading reinforcement characteristics of fractured coal under low confining pressure

**DOI:** 10.1038/s41598-023-38625-5

**Published:** 2023-07-26

**Authors:** Ying-ming Yang, Xue-bin Gu, Xin-jie Liu, Bai Lu, Xiao-jun Ding, Yong-qiang Zhao, Wei-long Zhang, Gang Liu

**Affiliations:** 1 State Key Laboratory of Water Resource Protection and Utilization in Coal Mining, Beijing, 102209 China; 2 National Energy Investment Group Co., Beijing, 100011 China; 3 College of Energy and Mining Engineering, Shandong University of Science and Technology, Qingdao, 266590 China; 4 China Shenhua Energy Company Limited, Beijing, 100011 China

**Keywords:** Energy science and technology, Engineering

## Abstract

To study the basic mechanical behavior and the reloading reinforcement characteristics of fractured coal, conventional triaxial loading tests with different fissure angle were first carried out. On this basis, conventional triaxial loading and unloading tests were conducted to investigate the reloading reinforcement characteristics of fractured coal. The results reveal that when the fissure angle was small, the stress–strain curve exhibited the multi-peak phenomena. As the fissure angle increased, the stress drop phenomenon in the peak region was weakened. With the increase of the fissure angle, the peak stress of the specimens increased and then decreased, while the elastic modulus showed an overall increasing trend, demonstrating the controlling effect of the crack angle. Meanwhile, the cyclic loading exhibited a certain enhancement effect on the strength of the fractured coals when the specimens was unloaded near the crack closure stress. The findings can provide a better understanding of the failure mechanism and reloading reinforcement characteristics of fractured coal.

## Introduction

Since coal reserves in the eastern part of China are decreasing, there is a growing proportion of coal being mined in the western part. Western China is rich in coal resources, with shallow coal seams and stable coal seam distribution, providing good mining conditions. The good mining conditions make the mining in this area characterized by fast pushing speed and high mining thickness^1–5^. This leads to the development of fractures in the overlying strata, and further induces problems such as groundwater loss, extensive surface subsidence and building damage^6–10^. Therefore, it is of practical significance to study the physical and mechanical properties and crack progradation behaviors of coal rock masses in western mining areas.

The physical and mechanical properties of rocks are the basis for theoretical analysis and numerical calculations, and are of great importance for guiding coal mining. Scholars have conducted numerous investigations on the basic mechanical properties of rocks in the western mining area, examining the effects of depth^11^, sediment age^12^, confining stress^13^, temperature^14^, and loading paths^15^. In addition, some scholars have studied the mechanical properties and damage mechanisms of coal and rock through acoustic emission (AE) methods^16–19^. With respect to the physical and mechanical characteristics of coal rock mass in western mining areas, there are many studies on the intact coal rocks, but relatively few studies on fractured coal rocks.

In the mining process, the bearing pressure of the coal body in front of the working face presents a cyclical change due to the periodic breakage of the overlying strata^20,21^. And it is important to study the mechanical characteristics of coal samples under the cyclic loading and unloading so as to reveal the strength characteristics of the coal body in the field. Scholars have examined the mechanical^22,23^, the damage^24,25^, and the energy characteristics^26,27^ of coal under cyclic loading and unloading. In the process of cyclic loading, some scholars found that cyclic loading has a certain enhancement effect on the coal strength^28^. And this phenomenon was also found in the tests on barite^29^, skarn^30^ and coal-rock composite specimens^31^. Therefore, it can provide an important reference value in the field to understand the strength characteristics of the coal specimens under loading and unloading conditions.

The previous studies have greatly contributed to the understanding of the basic mechanical behavior and crack evolution of fractured coal. However, in the western mine area, where the coal seam is shallow, it is characterized by low confining pressure. And there are fewer studies on the mechanical characteristics of fractured coal samples in western mining areas. Meanwhile, it is also important to study the reloading reinforcement characteristics of coal, where the reloading reinforcement characteristics refer to the strength enhancement of the specimen after reloading. Therefore, a typical mine in the western mining area was used as the research object, and the mechanical and failure characteristics of coal samples with different fissure angles were studied firstly. On this basis, loading and unloading tests of fractured coal samples under confining pressure of 0.3 MPa were carried out to investigate the reloading reinforcement characteristics of fractured coal.

## Experimental setup

### Specimen preparation

**Figure 1:**
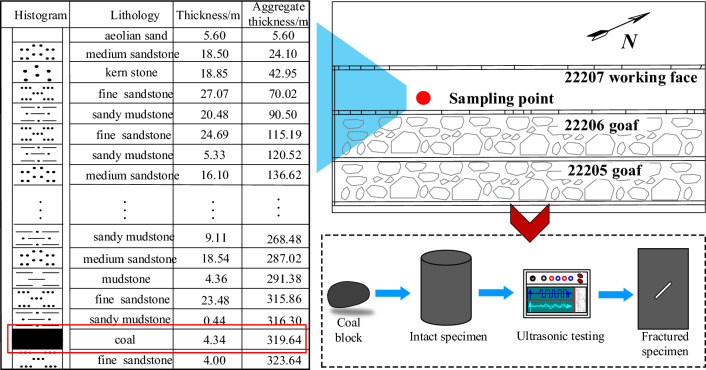
Sample location and production process.

The coal samples were collected from the Buertai coal mine in the Shendong mining region of western China; as shown in Fig. 1. First, a large coal block was selected and transported to the laboratory for processing. Cylindrical specimens with a height of 100 mm and a diameter of 50 mm were machined using cutters and grinders, ensuring that the non-parallelism of the two end faces was less than 0.05%. Subsequently, ultrasonic tests were performed and specimens with similar P-wave velocity and density were selected for subsequent tests. Finally, fractured specimens were prepared using wire-cutting equipment. The prefabricated fissure length was 20 mm, and dip angles were 0°, 15°, 30°, 45°, and 60°. Table 1 lists the physical and mechanical parameters of coal specimens.

**Table 1:** Physical and mechanical parameters of coal specimens.

Number	Density/g cm^−3^	P-wave velocity/m s	Uniaxial compressive stress/GPa	Elastic modulus/GPa
C-1	1.22	895.59	24.68	1.58
C-2	1.26	687.9	25.46	1.64
C-3	1.28	1104.65	27.61	1.95
Average	1.25	896.05	25.91	1.72

### Experimental system and test scheme

**Figure 2:**
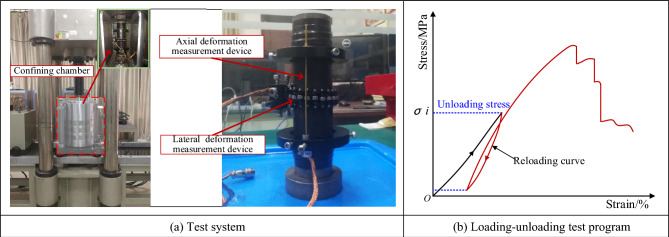
Test system and loading scheme.

The RLJW-2000 servo rock testing machine was used for the conventional triaxial loading test, and the system consists of a loading device and a monitoring device, as shown in Fig. 2a. The loading equipment can achieve the loading control of axial stress and confining pressure, and the monitoring device can monitor the axial deformation and lateral deformation.

1. Conventional triaxial loading test. To investigate the deformation and failure characteristics of fractured specimens with different fissure angle, a conventional triaxial loading test (with the confining pressure of 0.3 MPa and 3 MPa) was carried out for the fractured specimens of five different fissure angle types. In the test, the axial loading rate will be set to 0.25 mm/min.
2. Loading–unloading test under confining pressure of 0.3 MPa. To explore the reloading reinforcement characteristics of the fractured specimens, loading–unloading tests on fractured specimens were carried out under confining pressure of 0.3 MPa. Firstly, the specimen will be loaded to the unloaded stress (determined by conventional triaxial loading test), then unloaded to a lower stress level (500 N) and reloaded until the specimen is damaged, as shown in Fig. 2b. In the test, the axial loading rate is 0.25 mm/min.

## Test results of conventional triaxial loading

### Confining pressure of 0.3 MPa

#### Stress–strain curve

**Figure 3:**
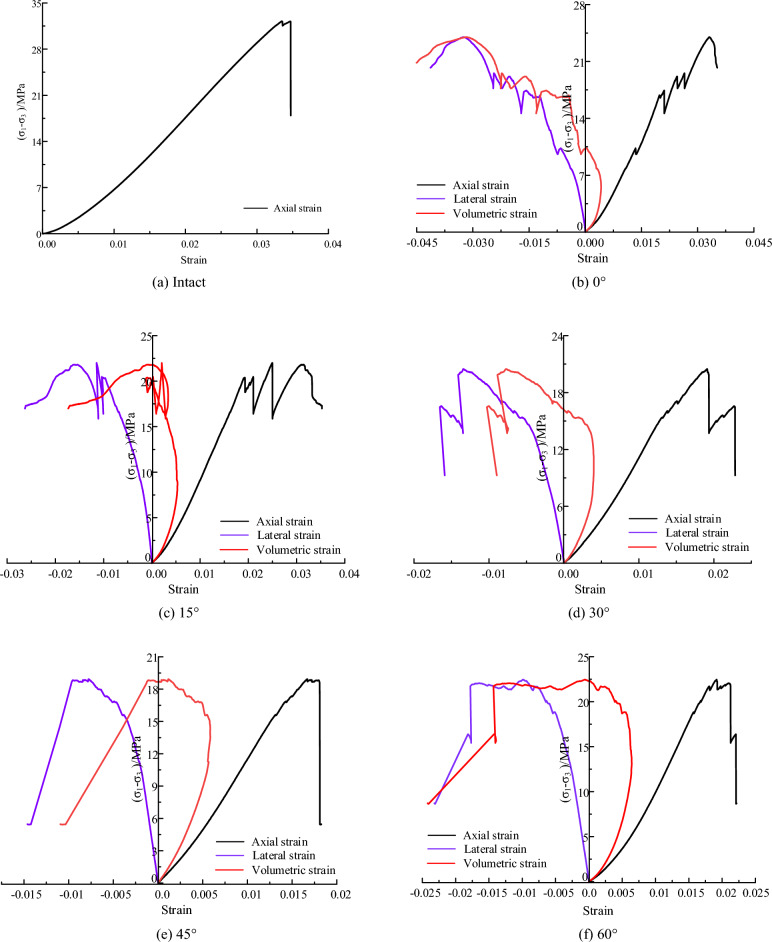
Stress–strain curves of fractured specimens under confining pressure of 0.3 MPa.

Figure 3 presents the stress–strain curves of the fractured coal specimens under confining stress of 0.3 MPa. The stress–strain curve can be divided into four typical stages: compaction, elastic deformation, crack unstable development and post-peak damage. Compared with fractured specimens, intact specimens experienced less stress drop before the peak stress, and the stress decreased rapidly when it reached the peak, exhibiting obvious brittleness characteristics.

As shown in Fig. 3, when the fissure angle was small, e.g., 0° and 15°, the stress-axial strain curve experienced several stress drops before the peak stress. When the peak stress was reached, the stress-volumetric strain curve showed a smooth falling trend, indicating a certain ductility characteristic. As the fissure angle increased, the stress-drop phenomenon weakened, such as 30°, 45° and 60°. And after reaching the peak stress, the stress-volumetric strain curve exhibited a rapid decreasing trend with a characteristic of brittle damage. Additionally, with the increase of the fissure angle, the axial stress corresponding to the maximum value of volumetric strain gradually increased. The axial stress increased from 5.6 to 10.4 MPa and 14.3 MPa when the fissure angle was increased from 0° to 30° and 45°.

#### Strength and deformation characteristics

**Figure 4:**
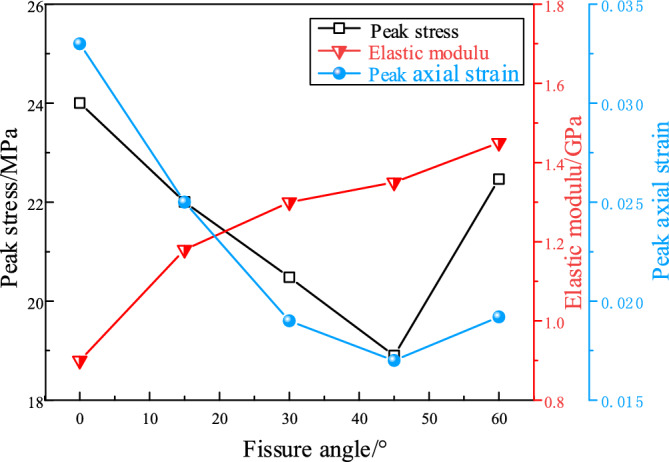
Mechanical parameters of fractured specimens under confining pressure of 0.3 MPa.

Figure 4 presents the variation curves of the mechanical parameters of fractured specimens under the confining pressure of 0.3 MPa. Peak stress, elastic modulus and peak axial strain were frequently used to characterize the strength and deformation ability of the specimen. Elastic modules is generally calculated with the gradient of the approximate linear part of the axial strain–stress curve, and the peak axial strain refers to the axial strain corresponding to the peak stress.

As shown in Fig. 4, with the increase of the fissure angle, the peak stress and peak axial strain of fractured specimens showed a trend of first decreasing and then increasing. For example, when the fissure angle increased from 0° to 45°, the peak stress decreased from 24 to 18.9 MPa, with a reduction rate of 21.3%. When the fissure increased to 60°, the peak stress raised to 22.5 MPa, with a growth rate of 19%. Meanwhile, the minimum strength of the specimens was observed when the fissure angle was 45°, which is consistent with the investigation by schloars^32^. This indicated that the existence of preformed fracture had a significant controlling effect on the strength and deformation of the specimen. As shown in Fig. 4, the elastic modulus showed an overall increasing trend with the increase of fissure angle. When the fissure angle increased from 0° to 30° and 60°, the elastic modulus increased from 0.9 to 1.3 GPa and 1.45 GPa, respectively. This is because the angle between the normal direction of the fissure and the loading direction gradually increased with the increase of the fissure angle. This leads to a tendency for specimens with high fissure angle to have smaller deformation under the same stress, causing an increase in the elastic modulus of the specimen.

#### Failure characteristics

**Figure 5:**
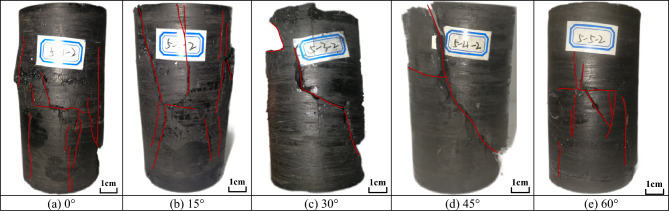
Failure mode of fractured specimens under confining pressure of 0.3 MPa.

Figure 5 illustrates the failure modes of specimens with different fissure angle under confining pressure of 0.3 MPa. As shown in Fig. 5a,b, when the fissure angle is 0° and 15°, multiple axial macroscopic cracks occurred on the surface of the specimen, indicating that tensile failure mainly occurred in the material. As shown in Fig. 5c,d, when the fissure angle is 30° and 45°, macroscopic cracks developed along the upper and lower tip of the preformed fissure, cutting the specimen tilted through, indicating that shear failure occurred in the specimen. As shown in Fig. 5e, when the fissure angle is 60°, multiple cracks appeared at the upper tip of the preformed fissure, showing X-shaped shear damage. Remarkably, there were axial cracks in the specimens as well, which indicated the occurrence of multiple damage modes within the specimens.

### Confining pressure of 3 MPa

#### Stress–strain curve

**Figure 6:**
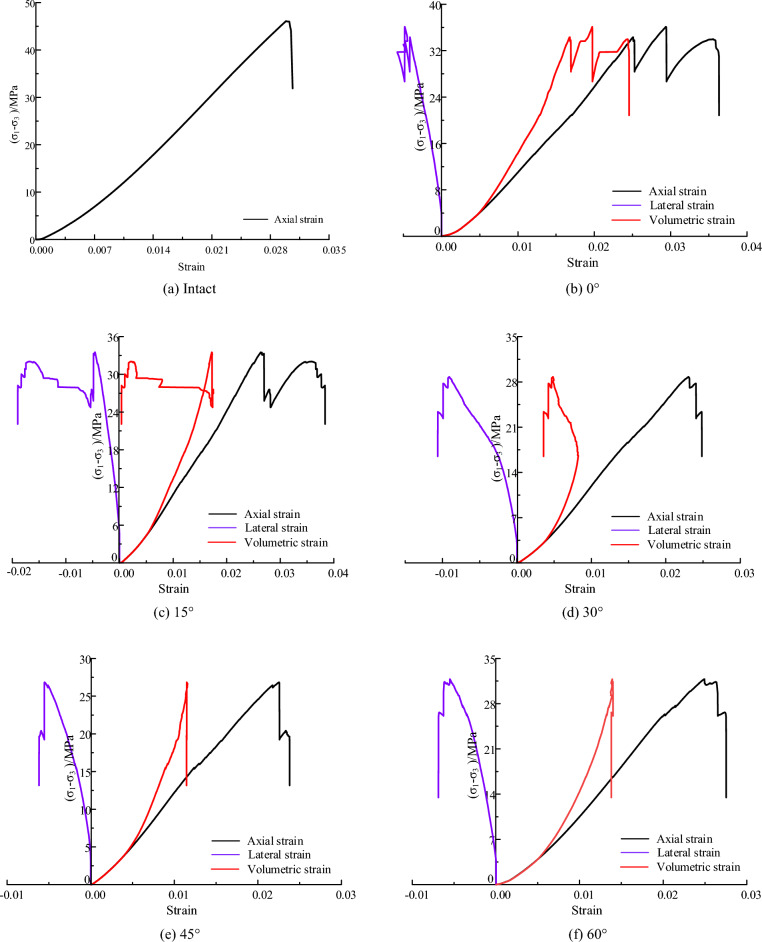
Stress–strain curves of fractured specimens under confining pressure of 3 MPa.

Figure 6 presents the stress–strain curves of the fractured coal specimens under confining stress of 3 MPa. When the fissure angle is small, the stress–strain curve in the peak region showed a phenomenon of ‘significant decrease-increase’. For example, when the fissure angle is 0° and 15°, the stress–strain curve exhibited two times and one time obvious 'stress decrease-rise' phenomenon respectively, and the magnitude of stress decrease can reach 10 MPa. And when the fissure angle is 0°, the lateral strain–stress curve presents irregular changes due to the sudden variation of stress in the peak region. With the increment of fissure angle, this stress drop phenomenon gradually becomes non-significant. When the fracture dip angle is 30° and 60°, a small stress drop was observed in the peak region, with a stress drop amplitude of about 1 MPa. This indicates that the fissure angle has a controlling effect on the shape of the stress curve.

#### Strength and deformation characteristics

**Figure 7:**
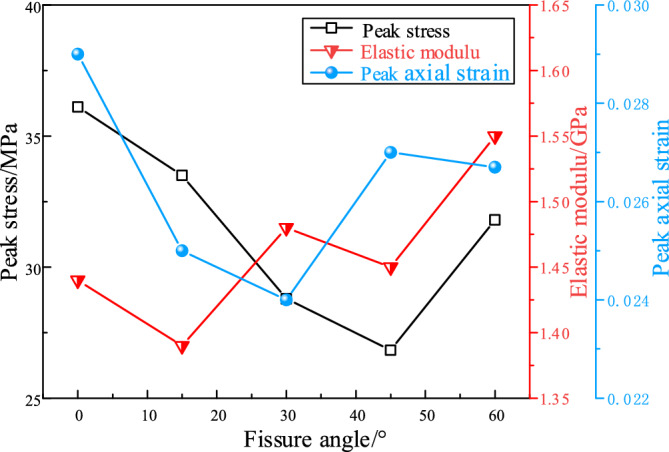
Mechanical parameters of fractured specimens under confining pressure of 3 MPa.

Figure 7 presents the variation curves of the mechanical parameters of fractured specimens under the confining pressure of 3 MPa. As shown in Fig. 7, with the increment of fissure angle, the peak stress showed a trend of decreasing and then increasing, which in consistent with the law under low confining pressure. For example, when the fissure angle increased from 0° to 45°, the peak stress reduced from 36.1 to 26.8 MPa. When the fracture dip angle increased to 60°, the peak stress rose to 31.8 MPa. With the increment of fissure angle, the elastic modulus shows an overall increasing trend. The elastic modulus was increased from 1.44 to 1.55 GPa when the fissure angle was increased from 0° to 60°. This indicates that the smaller the angle between the normal direction of the fissure and the loading direction, the greater the deformation of the specimen under the the same stress conditions.

#### Failure characteristics

**Figure 8:**
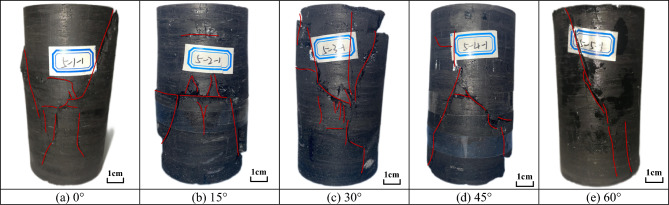
Failure mode of fractured specimens under confining pressure of 3 MPa.

Figure 8 illustrates the failure modes of specimens with different fissure angle under confining pressure of 3 MPa. As shown in Fig. 8a,b, when the fissure angle is 0°, multiple axial cracks were developed on the specimen surface, and in addition, an inclined macroscopic crack was generated at the top right of the specimen. Compared with the specimen with 0.3 MPa confining pressure, this indicates that the specimen turned into tensile-shear composite failure due to the increase of confining pressure. As shown in Fig. 8c,d, when the fissure angle is 30°, a crack was developed upward at the upper tip of the preformed fissure, and multiple macroscopic cracks were developed upward and downward at the lower tip of the fissure, respectively. When the fissure angle is 45°, an inclined crack was developed downward to the bottom of the specimen from the upper tip of the preformed fissure, and a crack was generated toward the right side of the specimen from the lower tip of the fissure. As shown in Fig. 8e, when the fissure angle is 60°, cracks developed upward and downward from the fissure tip to the specimen end face, respectively, indicating that the cracks mainly expanded along the preformed fissure.

## Discussion

### The determination of unloading stress

**Figure 9:**
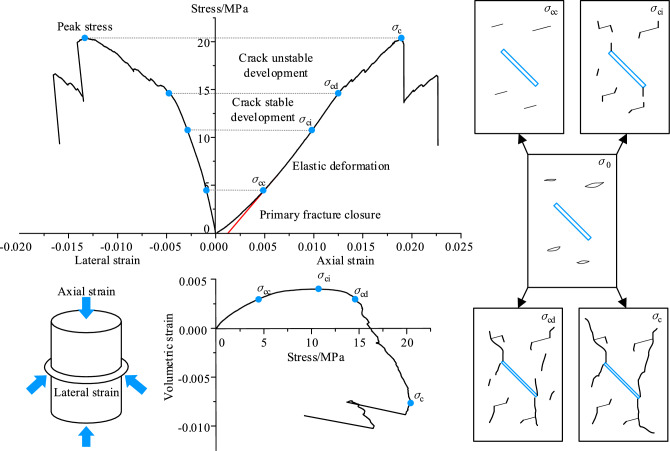
Stages division of stress–strain curve.

In the deformation and damage process of the coal, the closure of the primary fractures occur first, and then as the loading continues, new cracks will gradually be generated within its interior. As the stress level increased, cracks began to expand and accumulate, eventually forming macroscopic cracks and leading to the failure of the specimen. According to the characteristics of the stress–strain curve, the loading process can be divided into: primary fracture closure, elastic deformation, crack stable development, crack unstable development and post-peak phase^33^, as shown in Fig. 9.

To investigate the reloading reinforcement characteristics of the coal, the unloading stress point of the coal needs to be selected. Crack closure stress (CC, σ_cc_) refers to the stress corresponding to the closure of the internal primary fracture or pore, and the most common approach to determine the CC is the axial strain (AS) method^34^. The CC is identified as the stress level corresponding to the infection point at which the axial strain becomes linear after the initial nonlinear portion of the axial stress–strain curve, as shown in Fig. 9. Therefore, the stress value exceeding the CC of 1 MPa is chosen as the unloading point to ensure the complete compression of the internal primary fractures. The unloading stress values for different fissure angles under confining pressure of 0.3 MPa are shown in Table 2.

**Table 2:** Unloading stress values for different fissure angles under 0.3 MPa confining pressure.

Fissure angle/°	0°	15°	30°	45°	60°
Unloading stress/MPa	6.5	5.9	7	7.2	8

### Reloading reinforcement characteristics

**Figure 10:**
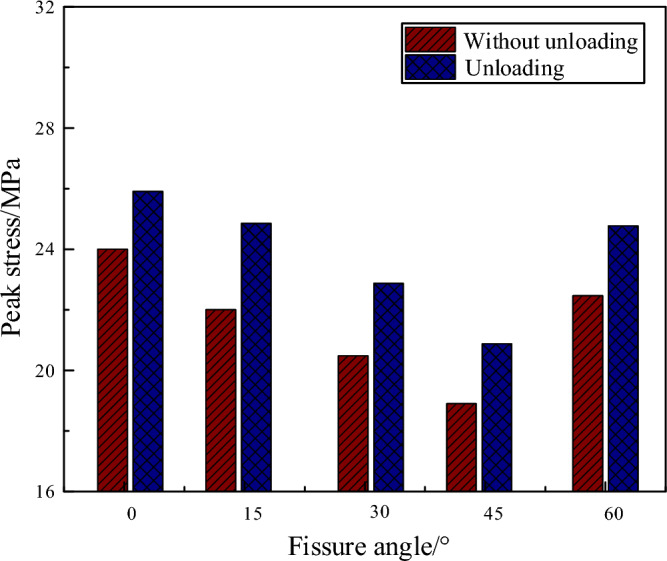
Peak stress of specimens with and without unloading under 0.3 MPa confining pressure.

Figure 10 presents the peak stress of specimens with and without unloading under 0.3 MPa confining pressure. As shown in Fig. 10, the strength of the reloading specimens was enhanced to a certain magnitude compared to that of the directly loaded specimens. When the fracture dip angle is 15°, the maximum stress increment was reached at 2.86 MPa. This finding is somewhat consistent with the conclusion reached by some scholars in studies of cyclic loading and unloading of rocks: the cyclic loading may lead to an increment in the peak strength of the specimen. Such as the study of coal-rock composite specimen by Zuo et al.^31^ and marble by You et al.^29^ and silica by Xu et al.^30^.

This paper conducted unloading-loading tests on the coal body and found that the strength of the coal body was improved in different degrees. This may be related to the unloading stress, and for the one-time cyclic loading test of the coal, when the unloading stress is moderate, the first loading would contribute to the closure of the microcracks inside the coal, and thus gradually increase the strength of the specimen. When the unloading stress is high, larger loads tend to cause new microcracks in the early stages of loading, leading to damage accumulation and decreased strength. Yang et al.^24^ investigated the cyclic loading for coal samples and showed that the strength of coal samples under cyclic loading did not exceed 81% of the uniaxial strength, which differs from the results of this paper. This may be related to the number of cycles, where multiple cycles cause a deterioration of the material, which in turn leads to a decrease in its strength.

## Conclusions

1. Compared with intact specimens, fractured specimens experienced more stress drop before the peak stress. When the fissure angle was small, the stress–strain curve exhibited the multi-peak phenomena. As the fissure angle increased, the stress drop phenomenon in the peak region was weakened.
2. With the increase of the fissure angle, the peak stress of the specimens increased and then decreased, while the elastic modulus showed an overall increasing trend, demonstrating the controlling effect of the crack angle.
3. When the fractured specimens were unloaded near the crack closure stress and then reloaded, there was a certain increase in the strength of the fractured specimens. This maybe related to the fact that a moderate first loading would help to close the microcracks inside the coal, and thus gradually increasing the strength of the specimen.

## Data Availability

The datasets used during the current study available from the corresponding author on reasonable request.
